# Editorial: Sarcopenia, Frailty and Nutrition in Liver Diseases

**DOI:** 10.3389/fnut.2022.929459

**Published:** 2022-06-08

**Authors:** Speranta Iacob, Susanne Beckebaum, Dan Lucian Dumitrascu, Liana Gheorghe

**Affiliations:** ^1^Carol Davila University of Medicine and Pharmacy, Bucharest, Romania; ^2^Center for Digestive Diseases and Liver Transplant, Fundeni Clinical Institute, Bucharest, Romania; ^3^Center of Excellence in Translational Medicine, Fundeni Clinical Institute, Bucharest, Romania; ^4^Department of Gastroenterology, Hepatology, Endocrinology und Clinical Infektiology, University Hospital Munster, Munster, Germany; ^5^2nd Department of Internal Medicine, Iuliu Hatieganu University of Medicine and Pharmacy, Cluj-Napoca, Romania

**Keywords:** sarcopenia, cirrhosis, fatty liver (see NAFLD), complications, chronic liver disease (CLD)

Sarcopenia is a multifactorial process, representing a progressive and diffuse loss of skeletal muscle mass, strength, and function, with a high prevalence in chronic liver diseases and a significant negative impact on survival, quality of life, and development of other complications of cirrhosis and post-liver transplant outcome (1).

This Research Topic aimed at collecting papers suitable to improve our knowledge about the interplay between malnutrition, sarcopenia, and frailty in chronic liver diseases as well as prevalence, risk factors, nutritional screening, outcome and prognosis, nutritional interventions, and exercise in non-alcoholic fatty liver disease (NAFLD), in advanced chronic liver diseases, and before and after liver transplantation (LT).

In this special e-book there are 13 papers covering the above mentioned aspects, the majority of them referring to liver cirrhosis and the others to NAFLD.

Due to the high prevalence of sarcopenia in cirrhotic patients (48.1%) (2) and the potential of sarcopenia in predicting diseases outcomes, identifying the risk factors of sarcopenia in patients with chronic liver diseases has become a critical clinical issue.

Hui et al. explored the relationship between sleep–wake disturbance and malnutrition risk in hospitalized patients with cirrhosis, and the Royal Free Hospital-Nutritional Prioritizing Tool (RFH-NPT) score was observed to be significantly higher in poor sleepers. Moreover, the Pittsburgh Sleep Quality Index has been demonstrated to be an independent risk factor positively correlated with RFH-NPT, meaning a high risk of malnutrition. The article by Wang et al. evaluated and confirmed that cirrhotic patients with a higher visceral to subcutaneous adipose tissue area ratio have a higher risk of malnutrition compared to patients with low subcutaneous adiposity or high visceral adiposity.

The study by Yin et al. showed that body mass index (BMI) cannot act as an independent prognostic predictor of liver cirrhosis, as already expected. However, in this paper, patients with cirrhosis and acute gastrointestinal bleeding may have a slightly lower short-term survival if they are overweight or obese. On the other hand, in the study by Geng et al., the fat mass and rate of the total body and trunk were significantly higher in Wilson disease (WD) patients, while the muscle and skeletal muscle mass of the total body and trunk were significantly lower in these patients. This finding may have implications for a higher rate of atherosclerosis and acute cardiovascular disease in WD patients. Also, the paper by Lv et al. concluded that the higher the serum albumin before continuous renal replacement therapy (CRRT), the lower the mortality of critically ill patients (a large proportion of cirrhotic patients) with acute kidney injury (the majority of them due to sepsis) treated with CRRT, and the higher the clearance efficiency of serum phosphorus.

Due to the association between sarcopenia in cirrhotic patients and increased mortality, sepsis complications, hyperammonemia, hepatic encephalopathy, and increased hospital length of stay after LT, multiple studies have focused on management strategies. Several potential therapeutic targets were identified: branched chain amino acid (BCAA) supplementation, myostatin inhibitors, and mitochondrial protective agents (2). The article by Ismaiel et al. of our collection explained how supplementation with BCAAs such as leucine, valine, and isoleucine could ameliorate protein synthesis, lipid and glucose metabolism, insulin resistance, and hepatocyte proliferation, and reduce oxidative stress in hepatocytes in liver cirrhosis. On the other hand, they emphasized the fact that administration timing, dose, and nutritional education regarding BCAA supplementation are considered essential factors that might lead to good or suboptimal results. We, as hepatologists, should be aware of this when prescribing BCAA to cirrhotic patients in order to ameliorate sarcopenia.

One of the papers from our collection (Topan et al.) prospectively demonstrated the high prevalence of sarcopenia in cirrhosis (57.2%) and the association between sarcopenia and portal hypertension-related complications (ascites), infectious complications (urinary tract infection and spontaneous bacterial peritonitis), and the risk of hepatocellular carcinoma (HCC). The first study (3) demonstrated that sarcopenia is a significant independent factor for HCC development in male patients with cirrhosis by multivariate competing risk analysis and could be explained by multiple pathways such as aging, physical inactivity, insulin resistance, vitamin D or zinc deficiency, and chronic inflammation.

The first study (Iacob et al.) evaluating the SarQoL^Ⓡ^ questionnaire in cirrhotic patients revealed that it can evaluate quality of life and, at the same time, identify subjects with sarcopenia and altered QoL. The SarQoL^Ⓡ^ questionnaire could identify patients that would benefit the most following a multidisciplinary approach and therapeutic interventions.

A very recent meta-analysis (4) showed that sarcopenia was independently associated with an ~2-fold higher risk of mortality in patients with cirrhosis, mortality that increased with greater severity, or longer durations of sarcopenia. The CONUT (Controlling Nutritional Status) score proved to be a reliable and easy-to-calculate tool in predicting the development of 3-month complications after LT that could be integrated in clinical practice, as proved in the article by Spoletini et al.. Malnutrition and immunologic compromise increase the risk of post-LT complications and this study showed a correlation between the CONUT score and the development of severe complications and 90-day and long-term mortality after LT.

The importance of these considerations is even more critical in light of the evolving epidemiology of LT candidates due to the increased prevalence of non-alcoholic steatohepatitis (NASH). A recent study investigating the relationship between frailty and cirrhosis etiology revealed that NASH patients were among the frailest category of LT candidates, justifying specific consideration to the liver functional reserve and malnourishment and immunologic impairment when a patient is transplanted (5).

That is why in our collection there is one article (Azevedo et al.) dedicated to the analysis of the complex relationship between sarcopenia and NAFLD. The authors discuss the key mechanisms linking NAFLD to sarcopenia and their clinical importance: the impact of body composition phenotypes on muscle morphology, the concept of sarcopenic obesity, the relationship between sarcopenia and the severity of the liver damage, and the future directions and existing gaps in the knowledge.

Vitamin D deficiency is among the well-known factors that influence the interplay between the muscle and liver together with insulin resistance, obesity, chronic low-grade inflammation, physical inactivity, aging, unhealthy diet composition, different hormonal changes, and oxidative stress. However, vitamin C deficiency could be a risk factor for NASH patients and its role as therapy in NAFLD is investigated in an article by Xie et al..

On the other hand, weight loss and lifestyle changes have a central role in the management of NAFLD. One mini review of our collection (Berkovic et al.) addresses the importance of physical activity in prevention, treatment, and its extrahepatic benefits in NAFLD.

*De novo* or recurrent NASH is increasingly reported following LT and up to one third of patients may develop recurrent bridging fibrosis/cirrhosis. Knowing the genetic and lifestyle risk factors for recurrent NAFLD (Iacob et al.) and trying to actively modify some of them is of utmost importance to prevent associated complications and retransplantation.

In conclusion, our e-book brings evidence to the fact that sarcopenia should be a component of initial evaluation of all cirrhotic patients regardless of severity or etiology of cirrhosis and should be regularly monitored and treated appropriately in order to ameliorate prognosis on the waiting list and after LT.

The continuous rise in the global prevalence of NAFLD, its progressive course, and associated consequences of sarcopenic obesity leads to the need of screening and fast implementation of preventive measures and treatment of NASH patients before and following LT.

## Author Contributions

SI, SB, DD, and LG wrote and reviewed the manuscript. All authors contributed to the article and approved the submitted version.

## Conflict of Interest

The authors declare that the research was conducted in the absence of any commercial or financial relationships that could be construed as a potential conflict of interest.

## Publisher's Note

All claims expressed in this article are solely those of the authors and do not necessarily represent those of their affiliated organizations, or those of the publisher, the editors and the reviewers. Any product that may be evaluated in this article, or claim that may be made by its manufacturer, is not guaranteed or endorsed by the publisher.
